# Atypical perceptual narrowing in prematurely born infants is associated with compromised language acquisition at 2 years of age

**DOI:** 10.1186/1471-2202-11-88

**Published:** 2010-07-30

**Authors:** Eira Jansson-Verkasalo, Timo Ruusuvirta, Minna Huotilainen, Paavo Alku, Elena Kushnerenko, Kalervo Suominen, Seppo Rytky, Mirja Luotonen, Tuula Kaukola, Uolevi Tolonen, Mikko Hallman

**Affiliations:** 1 Faculty of Humanities, Logopedics, University of Oulu, Finland; 2 Department of Clinical Neurophysiology, Neurocognitive Unit, Oulu University Hospital, Finland; 3 Cognitive Brain Research Unit, Institute for Behavioural Sciences, University of Helsinki, Finland; 4 Turku Institute for Advanced Studies, Centre for Cognitive Neuroscience, Department of Behavioural Sciences and Philosophy, University of Turku, Finland; 5 Finnish Centre of Excellence in Interdisciplinary Music Research, University of Jyväskylä, Finland; 6 Department of Signal Processing and Acoustics, Aalto University, Helsinki, Finland; 7 Institute for Research in Child Development, School of Psychology, UK; 8 Department of Phoniatrics, P.O. Box 25, Oulu University Hospital, Finland; 9 Department of Paediatrics, Oulu University and University Hospital, Finland

## Abstract

**Background:**

Early auditory experiences are a prerequisite for speech and language acquisition. In healthy children, phoneme discrimination abilities improve for native and degrade for unfamiliar, socially irrelevant phoneme contrasts between 6 and 12 months of age as the brain tunes itself to, and specializes in the native spoken language. This process is known as perceptual narrowing, and has been found to predict normal native language acquisition. Prematurely born infants are known to be at an elevated risk for later language problems, but it remains unclear whether these problems relate to early perceptual narrowing. To address this question, we investigated early neurophysiological phoneme discrimination abilities and later language skills in prematurely born infants and in healthy, full-term infants.

**Results:**

Our follow-up study shows for the first time that perceptual narrowing for non-native phoneme contrasts found in the healthy controls at 12 months was not observed in very prematurely born infants. An electric mismatch response of the brain indicated that whereas full-term infants gradually lost their ability to discriminate non-native phonemes from 6 to 12 months of age, prematurely born infants kept on this ability. Language performance tested at the age of 2 years showed a significant delay in the prematurely born group. Moreover, those infants who did not become specialized in native phonemes at the age of one year, performed worse in the communicative language test (MacArthur Communicative Development Inventories) at the age of two years. Thus, decline in sensitivity to non-native phonemes served as a predictor for further language development.

**Conclusion:**

Our data suggest that detrimental effects of prematurity on language skills are based on the low degree of specialization to native language early in development. Moreover, delayed or atypical perceptual narrowing was associated with slower language acquisition. The results hence suggest that language problems related to prematurity may partially originate already from this early tuning stage of language acquisition.

## Background

Basic auditory skills constitute a foundation for language development [1,2]. Healthy infants possess well-developed auditory capabilities from birth, allowing the perception of a wide range of auditory material, as indexed by behavioral [3-5] and electrophysiological methods of testing [6-8]. Perceptual development, however, undergoes a process of narrowing and specialization for almost all socially relevant stimuli - voices, faces, and speech sounds (for a review see ref. [9]). During the first months of their lives, infant's sensory systems are broadly tuned to any type of auditory material, and they are able to discriminate speech sounds regardless of whether these sounds belong to the surrounding adult language or not [10-12]. Language-specific discrimination abilities improve between 6 and 12 months of age for native [10], and decline for unfamiliar phoneme contrasts [13-15] as the brain tunes itself towards optimal perception of the native spoken language [13,14]. Several studies have suggested that improved native-phoneme discrimination skills are good predictors of later language performance [12,13,16], while the opposite has been observed to hold for the non-native phoneme discrimination. Atypically long lasting sensitivity to non-native speech contrasts may indicate poor brain commitment to a native language, and has been previously demonstrated to result in slower language development at the age of 2 years [12].

Most children develop language skills without effort, following a typical sequence of development. However, some children, including those born very prematurely, may have great difficulties in acquiring language [17-20]. Of those prematurely born children who survive, roughly half have language and learning disabilities [21,22], representing a growing public interventional and educational concern [21]. An atypical auditory processing has been demonstrated in prematurely born infants [23-25] which has been linked to atypical language [25] and cognitive development at school age [26]. However, there is currently no clear model which would provide information on stages of language, and auditory processing development in prematurely born infants through the first 2 years of life. Event-related potentials (ERPs) are a safe and reliable method to investigate language related auditory processing in infants long before their language production abilities can be assessed [27].

The ERP component called the mismatch negativity (MMN) is elicited by potentially discriminable changes in repeated auditory stimuli [28], and its latency and amplitude are correlated to behavioural discrimination accuracy [29,30]. Cheour et al. [10] found that amongst six-month-old monolingual Finnish infants, the non-native/õ/elicited higher MMN amplitudes than the native/ö/presumably due to the higher acoustic contrast compared to repeated native/e/. In contrast, at the age of one year, these infants showed a diminished MMN for the non-native/õ/but an increased MMN for the native/ö/, indicating long-term memory traces for native speech sounds formed between the ages of six months and one year [10]. These studies provide an electrophysiological evidence for neural tuning to familiar spoken material [10,13,14], and suggest it to be predictive of later language development [13,16].

The present study examined 1) the ability of prematurely born and full-term, healthy six-month-old infants to discriminate between native (rare native Finnish/ö/amongst repeated native/e/phoneme), and between native and non-native phonemes (rare non-native/õ/phonemes amongst repeated native/e/phoneme), as reflected by the MMN; and 2) the development of this ability during the subsequent period of six months; 3) language development at the ages of one and two years, and 3) an association of the development of neural discrimination ability with language abilities at two years of age.

We recorded the MMN from 11 very prematurely born monolingual infants (GA<32 weeks), and 13 full-term, healthy infants at the age of six months (± 1 week), and at the age of one year (± 1 week) to investigate whether these two groups of children differ from each other in their ability to discriminate between phonemes. Gestationally corrected age was used for the prematurely born infants.

Language skills (vocabulary development, the use of morphological structures in spoken language, and the mean length of the three longest utterances = MSL) of the full-term and prematurely born infants were assessed at the ages of one and two years using MacArthur Communicative Development Inventories (CDIs [31,32]).

## Results and discussion

**Figure 1:**
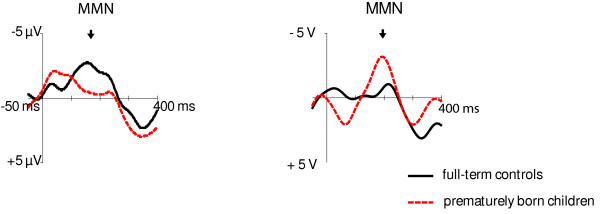
**Mismatch negativity (grand average, infrequent-frequent difference waveform) reflects the development of language specific memory traces (left-hemisphere C3 in the figure)**. Frequent phoneme was/e/, infrequent phoneme was non-native, Estonian/õ/. The MMN amplitude in response to non-native phoneme contrast diminished from the age of 6 months (on the left) to the age of 12 months (on the right) in the full-term controls, while in the prematurely born children this kind of reduction was not observed.

At the age of 6 months, there was no significant difference between the two groups in the MMN amplitude in response to non-native phoneme contrast (Table 1). Consistent with the theory of perceptual narrowing and previous studies [9,10,12], the amplitude of the MMN response to the non-native phoneme contrast, however, diminished between 6 and 12 months of age in full-term infants (F(1,24) = 3.288, P = 0.082; Figure 1; Table 1). In contrast, in the prematurely born children, this reduction was not observed, and at the age of one year, the MMN amplitude in response to the non-native phoneme contrast was significantly higher in prematurely born children than in the children born full-term (F(1,22) = 5.453, p = 0.029). Furthermore, there was a tendency for a Right-Left × Group interaction (F(1,22) = 4.125, P = 0.055) which was due to more enhanced MMN in the left hemisphere in children born premature than in the controls (Figure 1).

**Table 1:** MMN latencies and mean amplitudes in response to native and non-native phoneme contrasts at the ages of 6 and 12 months.

Condition and age	Latencies ms					Amplitudes μV				
	Premature Mean (SD)	Controls Mean (SD)	F	Df	P	Premature Mean (SD)	Controls Mean (SD)	F	Df	P
**Native phoneme**										
6 months	227 (40)	194 (29)	5,602	1.22	.027	-1.039 (2.12)	-.571 (1.24)	0,277	1.22	.604
12 months	200 (16)	188 (41)	0,777	1.22	.387	-.498 (2.58)	-.819 (2.79)	0,124	1.22	.728
**Non-native phoneme**										
6 months	216 (27)	197 (33)	2,216	1.22	.151	-.575 (2.15)	-.541 (1.24)	0,002	1.22	.962
12 months	198 (16)	199 (29)	0,020	1.22	.889	-1.061 (1.70)	+.323 (1.19)	5,453	1.22	.029

Thevalues represent the corresponding means and standard deviations over six electrodes. The P-values represent the result of the ANOVA analyses, indicating the significance of the between- group differences over all the electrodes.

At the age of 6 months, repeated measures ANOVA revealed no significant difference in the MMN amplitude in response to native phoneme contrasts between the two groups of infants. However, the MMN latency was significantly shorter in children born full-term than in children born premature (F(1,22) = 5.602, P = 0.027; Table 1 ), indicating faster discrimination of native phonemes by the former. In the children born premature the MMN latency tended to shorten between 6 and 12 months of age (F(2,20) = 4.178, P = 0.054) while no such changes in the MMN latency were observed in the children born full-term. At the age of one year, no significant difference in the MMN latency was found between prematurely born and full-term infants anymore. Neither was there any significant difference in the MMN amplitude. The results indicate that the formation of long-term memory traces for native phonemes was already well-developed by the age of 6 months in the children born full-term, while in the very prematurely born children, the native-language phoneme discrimination still continued to develop up to the age of 12 months.

The language measures at 12 months did not yield any significant differences between the groups. At the age of two years, however, the prematurely born children produced significantly less words (F(1,19) = 8.522, P = 0.009), and had shorter MSL, as indexed by the number of morphemes produced in sentences (F(1,19) = 6.819, P = 0.017) than the full-term children. Furthermore, the morphological structures of the sentences were less developed in the prematurely born children than in the full-term children (F(1,19) = 5.270, P = 0.033), as also reported in earlier studies [17].

**Figure 2:**
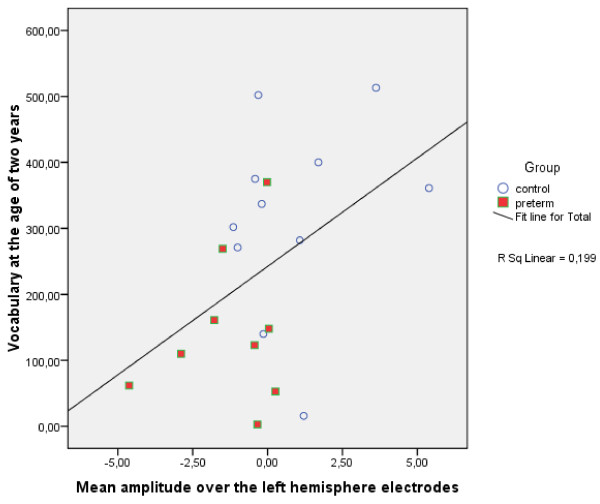
**Correlation between the MMN mean amplitude over the left hemisphere electrodes (F3, C3, P3) in response to non-native phoneme at the age of 1 year and the number of words produced by the children at the age of 2 years**. Correlations are over both groups. Horizontal line is the MMN mean amplitude over the left hemisphere electrodes, vertical line the number of words produced by each child as shown by the MacArthur Communicative Development Inventories (CDI). The result showed that the more negative the mean amplitude indicating better discrimination of non-native phoneme, the less the child produced words at the age of two years.

To explore whether phonetic discrimination abilities, as reflected by the MMN, are associated with behaviourally measured language skills, as reflected by the CDI, a correlation analysis was performed. The correlation analysis revealed that the larger was the MMN amplitude in response to the non-native phoneme at the age of one year, the less the child produced words (r^2 ^0,199, P = 0.048; Figure 2), the less developed the morphology (r^2 ^0,268, P = 0.019), and the shorter the MSL was (r^2 ^0.376, P = 0.004) at the age of two years. The findings indicate that those infants who did not acquire neural long-term representations specific to native-language phonemes at the age of one year, performed also worse in all subtests of the CDI language test at the age of two years.

In this study we examined the association between native and non-native phonetic discrimination in a group of monolingual, full-term, healthy children, and in a group of very prematurely born children. Consistent with the previous studies [14] we showed that long-term memory traces for native phonemes are well-developed in infants born full-term by the age of 6 months. In contrast, prematurely born infants continued to develop this ability up to the age of one year, as indexed by the shortening of the MMN latency. The most striking finding in these children, however, was that the discrimination of non-native phoneme contrasts strengthend from 6 to 12 months of age, which was negatively associated with several measures of linguistic skills at the age of two years. Thus, the prematurely born infants appeared to continue to maintain their ability to discriminate accurately non-native phonemes at the age of one year. In contrast, children born full-term showed a decrease in their ability to discriminate non-native vowels, as is typical for normal development [14].

The theory of native-language neural commitment [14] suggests that normal language development involves plastic changes while the brain tunes itself to native phonemes at the expense of its ability to process unfamiliar phonemes. Our study shows that this tuning is delayed or atypical in prematurely born infants. In prematurely born infants, an acoustically larger but non-native contrast evoked a larger brain response, suggesting that lower-level processing of physical acoustic characteristics is still dominating over language-specific processing at the age of one year. The result is in accordance with previous studies indicating a higher sensitivity to larger acoustic contrasts in infants born premature [24]. Thus, the finding of the present study suggests that language problems in prematurely born children may partially originate already from this early tuning stage of language acquisition.

There is a possibility that children born premature have not only a deficit in perceptual narrowing but also a more general auditory processing deficit. Further studies are needed to investigate the specificity of this deficit to native and non-native vowel contrasts. A new method, optima or multifeature paradigm [33], enables the use of different deviants in the same paradigm, and would therefore be a valuable method to define auditory processing deficits in prematurely born children in more detail. It would also be interesting to follow-up the same group of children from infancy to later age to investigate whether children born premature and showing deficits either in auditory processing or language development ever catch up their peers.

Premature birth constitutes a set of health risks for the infant. Minor but common deficits, like atypical auditory processing and slight delays in language development, are in most cases not diagnosed. Nevertheless these deficits may lead to later language and learning disabilities. Information provided by this study might be crucial for the early identification of infants at-risk for later language and learning deficits. Thus, prematurely born infants would benefit from information concerning their early language-related brain plasticity for early identification of infants at-risk for later language and learning deficits, and for introducing them to early interventions always when needed.

## Conclusions

The results of our follow-up study show for the first time that perceptual narrowing for non-native phoneme contrasts found in the healthy controls at the age of one year was not encountered in very prematurely born infants. Moreover, our results showed that this delayed or atypical perceptual narrowing was associated with slower language acquisition. The results hence suggest that language problems in prematurely born children may partially originate already from this early tuning stage of language acquisition. Further studies are, however, needed to investigate whether this deficit is specific to perceptual narrowing or whether it is related to a more general auditory processing deficit and whether these children ever catch up their peers.

## Methods

### Stimuli

The stimuli were Finnish vowels /e/ (frequent) and /ö/ (infrequent) as well as Estonian /õ/ (infrequent) which is non-native to Finnish infants. The acoustic difference between the /e/ and /õ/ is bigger than /e/ and /ö/. The stimuli were presented through closed-type headphones (Please, see detailed information of the stimuli ref. [10]).

### EEG measurements

The measurements were performed in an acoustically and electrically shielded room. During the measurements, the infants were seated in a safety seat, and an assistant entertained the infant with soundless toys to keep the infant relaxed and satisfied during the experiment. The stimuli, 400 ms (with 10 ms rise and fall times) were binaurally presented through headphones (75 dB SPL) with a 650 ms sound-onset asynchrony from onset to onset. The electroencephalogram (on-line bandpass 0.05-70 Hz, sampling rate 500 Hz) was recorded at the F4, C4, P4 (right hemisphere) and F3, C3, P3 (left hemisphere) sites, according to the international 10-20 system, using NeuroScan 4.0 amplifiers and software. Electro-ocular activity was recorded with two electrodes, one attached below the outer cantus of the left eye, and the other above the outer cantus of the right eye. Epochs (-100 to 500 ms) exceeding 200 μV in amplitude at any electrode were omitted from averaging. During the recording, the reference electrode was at FCz. After averaging, the data was re-referenced to the ipsilateral mastoids. Frequencies higher than 15 or lower than 1 Hz were digitally filtered out off-line. The MMN was analyzed from the difference waveform (the response elicited by the standard stimulus subtracted from that elicited by the deviant stimulus). The MMN was identified as the most negative peak within the time window of 150-300. The mean amplitudes were measured from the difference waves with a 100 ms window centered at the peak amplitudes of these waves (± 50 ms). The between group differences (prematurely born children, full-term children) were tested separately for native and non-native phonemes at six electrodes using repeated measures ANOVA with Group as a between-subject factor and Hemisphere [Right (F4, C4, P4) & Left (F3, C3, P3)] × Anterior-Posterior [(Frontal (F3, F4) & Central (C3, C4) & Parietal (P3, P4)] as within-subject factors. Developmental change for each phoneme were performed separately for the prematurely-born infants' data and for the control data by the ANOVA with Age [6 & 12 months] as a between-subject factor and Hemisphere [Right (F4, C4, P4) & Left (F3, C3, P3)] × Anterior-Posterior [(Frontal (F3, F4) & Central (C3, C4) & Parietal (P3, P4)] as within-subject factors. The Huynh-Feldt Correction was applied when appropriate.

### Behavioural measurements

Language development, comprehension and production, was assessed at the ages of one and two years by using the MacArthur Communicative Development Inventories (CDIs [31]) which is a questionnaire designed to assess both language comprehension and production in children between ages 8-30 months. In the CDI Words and Gestures (for 8-16 month old infants, used here for 12 month olds), parents document the child's understanding of early vocabulary items separated into semantic categories such as animal names, household items, and action words. Parents report the words understood and the words used by the infant, and the forms yield separate indexes of understanding and production. In the CDI Words and Sentences (for 16-30 month old children, used here for 24 month olds), parents report the child's production and use of words divided into semantic categories. In addition, the parents are asked to answer, whether the child uses certain morphological structures in spoken language (like plural and verb forms), and provide written examples of the child's three longest sentences or words that the child has used. In this study the CDI questionnaire was sent to parents two weeks before the MMN measurements were performed at the age of 12 months, and the CDI questionnaire was received from them at the MMN measurements. At the age of 24 months, questionnaires were sent and received from the parents by mail. Questionnaires were not received from parents of two prematurely born children and two control children. A One-Way ANOVA was used to compare the language test results between the groups, and correlations between the MMN and CDI values were tested using Spearman's Correlation Coefficients.

### Subjects

13 full-term (average gestational age: 40 weeks; SD 1,3 weeks; birth weight average 3720 g, SD 530 g) children and 11 children born very prematurely (gestational age <32 weeks, average 29 weeks, SD 1.7 weeks; birth weight average 1291 g, SD 411 g) served as participants. The postnatal ages were calculated on the basis of the post conception age of 40 weeks. All the children had normal hearing in each ear, as indexed by transient otoacoustic emissions, and normal auditory brainstem responses in prematurely born children at the stimulus level of 40 dB. The study was approved by the ethical committee of Oulu University Hospital.

## Authors' contributions

EJV, TK and MH conceived and designed the study. UT, TK and ML coordinated and performed hearing measurements. EJV and PA prepared the stimuli. EJV, KS, SR coordinated and performed the EEG recordings. EJV measured language development. EJV and EK performed data analysis. EJV, EK, TR and MH interpreted the results. All authors participated in the writing process and read and accepted the final version of the manuscript.
